# Engaging stakeholders: lessons from the use of participatory tools for improving maternal and child care health services

**DOI:** 10.1186/s12961-017-0271-z

**Published:** 2017-12-28

**Authors:** Elizabeth Ekirapa-Kiracho, Upasona Ghosh, Rittika Brahmachari, Ligia Paina

**Affiliations:** 1 Department of Health Policy, Planning, and Management, Makerere University School of Public Health, New Mulago Hospital Complex, Kampala, Uganda; 2 IIHMR University, 1 Prabhu Dayal Marg, Sanganer, Jaipur, 302029 India; 3 Department of International Health, Johns Hopkins Bloomberg School of Public Health, 615 N. Wolfe Street, Baltimore, MD 21205 United States of America

**Keywords:** Participatory, Stakeholders, Network analysis, Engagement

## Abstract

**Background:**

Effective stakeholder engagement in research and implementation is important for improving the development and implementation of policies and programmes. A varied number of tools have been employed for stakeholder engagement. In this paper, we discuss two participatory methods for engaging with stakeholders – participatory social network analysis (PSNA) and participatory impact pathways analysis (PIPA). Based on our experience, we derive lessons about when and how to apply these tools.

**Methods:**

This paper was informed by a review of project reports and documents in addition to reflection meetings with the researchers who applied the tools. These reports were synthesised and used to make thick descriptions of the applications of the methods while highlighting key lessons.

**Results:**

PSNA and PIPA both allowed a deep understanding of how the system actors are interconnected and how they influence maternal health and maternal healthcare services. The findings from the PSNA provided guidance on how stakeholders of a health system are interconnected and how they can stimulate more positive interaction between the stakeholders by exposing existing gaps. The PIPA meeting enabled the participants to envision how they could expand their networks and resources by mentally thinking about the contributions that they could make to the project. The processes that were considered critical for successful application of the tools and achievement of outcomes included training of facilitators, language used during the facilitation, the number of times the tool is applied, length of the tools, pretesting of the tools, and use of quantitative and qualitative methods.

**Conclusions:**

Whereas both tools allowed the identification of stakeholders and provided a deeper understanding of the type of networks and dynamics within the network, PIPA had a higher potential for promoting collaboration between stakeholders, likely due to allowing interaction between them. Additionally, it was implemented within a participatory action research project. PIPA also allowed participatory evaluation of the project from the perspective of the community. This paper provides lessons about the use of these participatory tools.

**Electronic supplementary material:**

The online version of this article (doi:10.1186/s12961-017-0271-z) contains supplementary material, which is available to authorized users.

## Background

Effective stakeholder engagement in research and implementation is important for improving the development and implementation of policies and programmes [1–4]. We define stakeholders as individuals, groups or organisations who have the potential to influence or who may be influenced by particular actions or aims [3, 5]. Stakeholders are not uniform, but vary in each context by their available resources, their position and their interests. Consequently, reasons for engaging them, and their engagement levels with a project, may differ. Arnstein [6] proposed eight levels of stakeholder participation, wherein the first (manipulation) and second level (therapy) allow no participation at all, while the third (informing), fourth (consulting) and fifth (placation) allow forms of tokenism in which stakeholders are informed of issues and their views are sought (fourth and fifth), but decisions are still made by those who hold power. Finally, in the sixth (partnership), seventh (delegated control) and eighth (citizen control) levels, shared decision-making and increasing levels of control are given to the stakeholders.

Overall, the process of stakeholder engagement can be mutually beneficial. Stakeholders may choose to engage with researchers because the research project might directly affect individual stakeholder interests, the engagement process might have financial incentives or benefits, or the engagement may lead to outcomes or outputs that benefit the general population [3]. Researchers and project implementers, on the other hand, may have slightly different reasons for engaging stakeholders, including to understand the power, interests, perspectives, values, behaviours and opinions of stakeholders, to understand how change happens in different contexts and among different individuals, to build the capacity of local stakeholders by creating a learning process and developing leaders and teams, to create a stimulus for change, to promote local ownership, and to assess the effect of a programme [7–11].

According to Durham [11], when choosing a method for engaging with stakeholders, it is important to consider the aim of the engagement, the resources available and the expectations of stakeholders. In practice, researchers have employed various tools to engage stakeholders. Provision of information to stakeholders has often been done through simple stakeholder workshops or meetings. Alternatively, consultation of stakeholders about their interests, needs, relationships, perceived benefits of a project, or about drivers of change has been performed through a range of methods that include most significant change, participatory evaluation, positive deviance approach and beneficiary assessment [2, 4, 7, 8, 10, 12]. For higher levels of engagement, participatory mapping and/or participatory social network analysis (PSNA) can be used to facilitate stakeholder involvement. Finally, tools such as participatory impact pathways analysis (PIPA) and approaches such as participatory action research are used by researchers to develop active partnerships and stakeholder engagement in project decision-making.

Participatory approaches are increasingly being advocated for because they give stakeholders a voice and allow them to table their concerns, as well as improving the identification of local problems and suggestions of feasible solutions and promoting the uptake of local solutions [13–15]. However, participatory approaches differ in the extent to which they involve the community in decision-making and hence in the extent to which they empower the community to address problems [14]. Approaches that are simply used to inform the community and stakeholders about what will be done, or that are used to facilitate community involvement in predetermined activities without shared decision-making are examples of passive community participation that generally tend not to empower the community, while those that allow the community to identify what their problem is and to get involved in identifying solutions for these problems are examples of active community participation. The latter empower the community to deal with not only their current problems, but also their future problems [13–16].

The use of many of these methods is still in its infancy, especially in low-income countries [17, 18]. In this paper, we discuss two participatory methods for engaging with stakeholders – PSNA and PIPA, which we have adapted and used to engage stakeholders as part of our work in the Future Health Systems (FHS) project in India and Uganda, respectively. Based on our experience, we derive lessons about when and how to apply these tools. Our work adds to the existing literature that summarises practical experiences with the use of these tools, highlighting the applicability and limitations of using the methods in different contexts.

### Overview of the tools and the context in which they were applied

Social network analysis (SNA) has been defined as a tool that allows the mapping and measuring of relationships and flows between people, groups, organisations or other information/knowledge processing entities [19, 20]. Furthermore, it provides an opportunity to compare formal and informal information flows. Such information can guide the planning and implementation of new interventions [17].

According to Blanchet [17], there are three main stages in SNA, namely (1) identification and description of the actors, (2) characterising the relationships between the actors, and (3) analysing the structure and pattern of the network. PSNA follows the three outlined stages, but also adds the use of participatory approaches that permit more interaction between the researchers and the participants and allows for feedback of results to stakeholders [21, 22]. These results can then be used to identify issues that need to be resolved – by so doing it provides a catalyst for change [21, 23]. However, for this to happen, there must be a level of trust between the researchers and the participants so as to allow free discussion [21]. In addition, the participants need to have the willingness and ability to solve any issues that they feel warrant their attention [23].

PSNA was applied in the Indian Sundarbans – the world’s largest mangrove delta – as part of a knowledge intervention aimed at engaging different stakeholders through knowledge creation, dissemination and effective up-take of knowledge regarding child health in the Sundarbans to inform and influence existing health policies in the region. The Sundarbans region is characterised by poverty, with frequent climatic events, which often lead to massive destruction of the already poor infrastructure, leaving behind displaced families with insufficient food and low productivity of the land for cultivation and ponds for fishing. This situation has led to migration of males in search of alternative livelihood, creating significant numbers of women-headed households. Furthermore, the child health status is poor, with chronic malnutrition and a high burden of communicable diseases [24]. Public health service delivery options are either absent or non-functional. Although non-governmental organisations (NGOs) provide some services, they cover only a limited area. Consequently, the gaps in health service delivery are filled by numerous Informal Healthcare Providers (IHPs), who practice modern medicine without any formal training or authorisation, locally referred to as village doctors or quacks.

PIPA is a relatively new planning, monitoring and evaluation tool designed to help the people involved in a project, programme or organisation work out how they will achieve their goals and impact [18, 25, 26]. PIPA analyses project impact through the use of problem trees and network pathways. The problem trees utilise linear logic that shows how the problems solved by the project eventually contribute to solving other related problems, achieving the programme goal. On the other hand, the network pathways show how the actions and interrelationships between different actors contribute to creating an enabling environment to solve the problems identified [18]. PIPA involves five distinct steps that include construction of problem trees, visioning, developing network perspectives, and defining an outcome logic model and an impact model. PIPA is usually implemented through 2- to 3-day workshops. The sessions are conducted through group meetings that comprise 4–6 stakeholders with a total of 3–6 groups. The workshops may be done at the beginning, middle and end of a project. However, different implementers have used it at different time points in their study. Alternatively, smaller reflection meetings can also be held to monitor progress, for example, every 6 months. These meetings provide an opportunity for learning and hence can provide a springboard for action research. In addition, for follow-up reflection meetings, linking the PIPA meeting to other technical or administrative meetings seemed to work better [18].

Some of the benefits that have been attributed to the use of PIPA include providing mutual understanding about intervention logic and the potential for achieving impact, an opportunity for ex ante impact assessment and a hypothesis for post ante impact assessment, in addition to providing a framework and design that enhances implementation that is aligned to the project/programme plans with room for learning during the monitoring and evaluation process. It can also promote collaboration between different programmes by making existing opportunities explicit [18].

The PIPA tool was implemented in three rural districts in Uganda (Kamuli, Kibuku and Pallisa), as part of a project that aimed to increase the number of births attended by skilled attendants. These districts have a high maternal and neonatal mortality rate comparable to that of the rest of the country (maternal mortality rate of 438 per 100,000 live births, neonatal mortality rate 27 per 1000 live births) [27]. In Uganda, the uptake of cost effective interventions that can reduce this maternal and neonatal mortality has been limited by factors such as poor maternal and newborn care practices, poor healthcare seeking behaviour, lack of financial means, inadequate infrastructure, and the existence of few overworked and poorly motivated health workers [28–31]. The Ugandan FHS project, MANIFEST (Maternal and Neonatal Implementation for Equitable Systems), focused on addressing problems related to inadequate knowledge about maternal and neonatal healthcare (MNH) practices, birth preparedness, poor access to emergency and routine transport, and poor quality of care at health facilities. Community mobilisation strategies supported locally organised, financed and monitored transport systems. Linkages between the community and the health facility were improved by using community health workers, who in Uganda are called Village Health Teams (VHTs). Quality of care improvements were stimulated using only non-financial incentives, which included training of health workers, mentorship, supportive supervision and recognition awards. The project was implemented using a participatory action research approach. PIPA was therefore seen as a method that would allow participatory monitoring of impact not only through the eyes of the researchers, but also through those of the community, who were both participants and implementers in this project.

## Methods

This paper was informed by a review of project reports and documents in addition to three reflection meetings. The documents that were reviewed include project proposals that describe how the method was applied, as well as research team reports summarising the stakeholder engagement activities. Research team members who were involved in using the methods in India and Uganda attended the first two meetings (one meeting in each country). The third meeting was used to clarify any remaining issues. The lead author and a member of the India team attended this meeting. The meetings were structured around why the method was selected, how the method was applied, training, methods used to collect data, resource requirements, how the method was adapted and key lessons learnt while applying the method [11]. During the meetings, we took notes and also recorded the discussions. These notes where then analysed by two of the authors of this paper to identify key themes, which have been presented herein. Research team reflections on using these tools for stakeholder engagement were synthesised using the conceptual framework presented below. The involvement of an author who was not directly involved in the research project and data collection for PIPA ensured that an objective perspective was maintained during the writing of the paper.

### Conceptual framework

The framework was developed based on existing literature about the purpose, process and outcomes of stakeholder engagement processes. It highlights the fact that the purpose of the engagement determines how the engagement is done, while the process of engagement influences the outcomes of the engagement process [11].

**Fig. 1:**
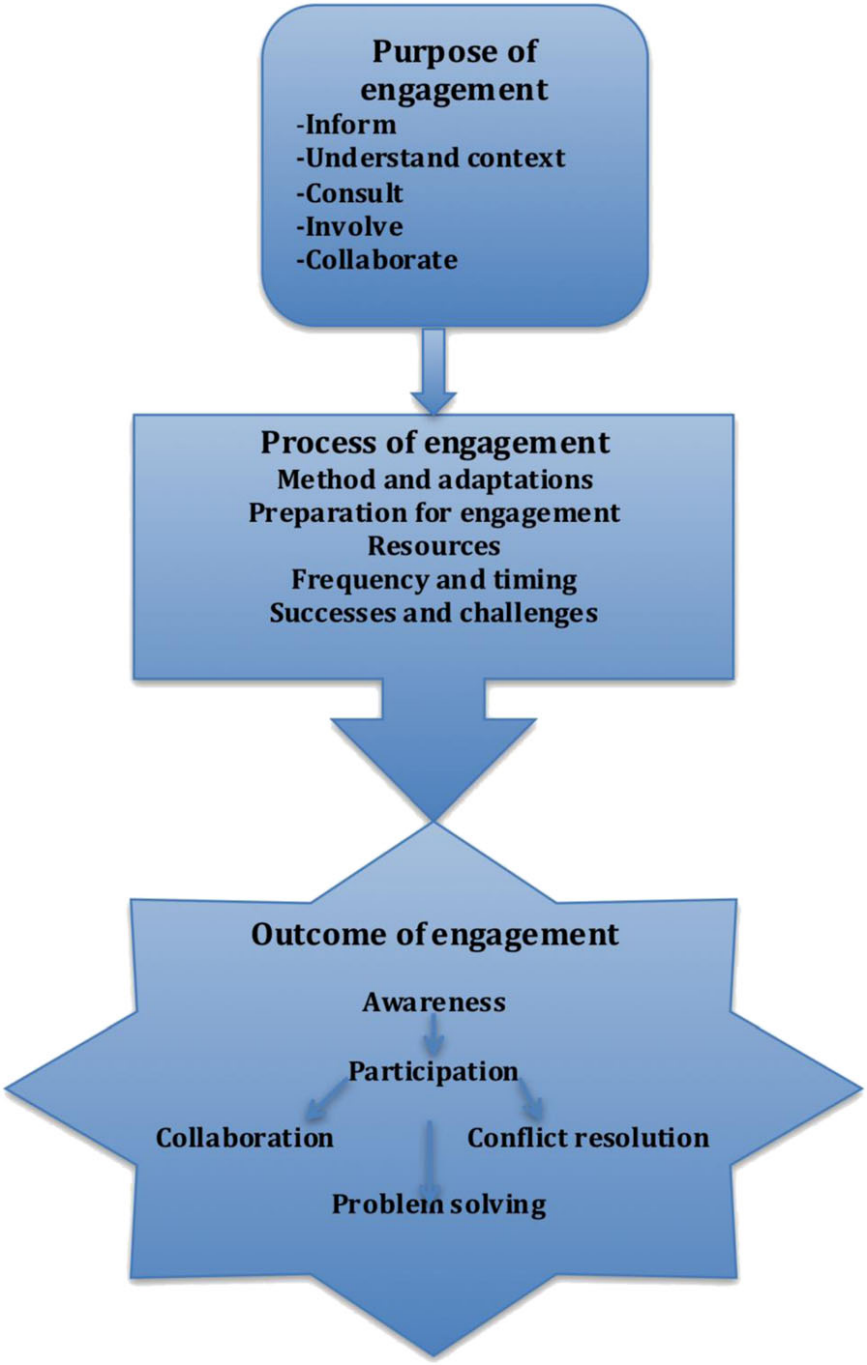
Conceptual framework

Based on this framework, we explore how the purpose for the engagement influenced the choice of tools applied. Furthermore, we explore how the application of PIPA and PSNA influenced the process and outcomes of the engagement (Fig. 1).

## Results

In this section, we summarise results for each of PSNA and PIPA, beginning with the purpose of engagement and why the tools were selected, as well as the processes involved in applying the method and outcomes of the engagement.

### Applying PSNA in the Sundarbans

#### Purpose of the engagement and why PSNA was selected

We engaged the stakeholders in the Sundarbans because we wanted to identify the type and nature of linkages that exist between the demand (mothers with children aged 0-6 years) and supply side (informal healthcare providers), and to understand how the linkages were formed within the given social context. The knowledge intervention programme aimed at generating evidence, disseminating it and building the capacity of the stakeholders to use the evidence to take the required actions. Hence, it was important to identify the crucial actors within the existing health system and their connection with the other actors who can act as agents of change. Furthermore, the knowledge intervention was implemented in a participatory manner to ensure better representation of the stakeholders. Therefore, PSNA was selected as it allows an in-depth understanding of the nature and the genesis of social ties from the stakeholders’ perspective and provides an understanding of the dynamics of the network connection of the health system.

### Process of engagement

#### Preparation for the engagement

Preparation for the engagement revolved around identifying a suitable location to conduct the activities, purchasing the necessary resources, identifying the researchers who would conduct the activity, and identifying and informing respondents who were to be included. The team decided that meetings would be held in the homes and workplaces of the respondents. Permission to hold the meetings in these venues was therefore sought from the respondents themselves through a signed consent form in vernacular language. The researchers were selected from the existing pool of FHS researchers within the India team on the basis of their personal interest in undertaking SNA studies and social science background.

#### Resources required

The key resources that were required included a venue, researchers to conduct the activity, instruments for data collection, and stationery such as blank chart paper, colour pens, sticky notes and a recorder. The data collection instrument was comprised of a semi-structured questionnaire to collect respondents’ identification and demographic data. A guideline for probing during network drawing was also prepared. An audio-recorder was used to capture respondents’ comments.

#### Training of facilitators

Two researchers and one research assistant facilitated the research work. The facilitators had prior knowledge and experience about qualitative research and this facilitated their understanding of the local context and nuances of the application of the participatory approaches. The training that was conducted focused on understanding the tools and how to administer them in a participatory manner, as well as on detailing how the questions would be asked and how further details would be probed for and how to guide the respondents to draw their network map. The training was participatory and facilitated by the lead researcher, and had a duration of one and a half months. During this period, two pilots were undertaken, which allowed the team to refine the data collection questions and process.

#### Application of the method

The team implemented two consecutive egocentric SNA studies in two blocks of Indian Sundarbans to explore the connectedness of one demand side stakeholder, i.e. the mothers of the children aged 0–6 years, and a supply side stakeholder, i.e. the IHPs. A total of 20 mothers were selected, of whom 10 were women with migrant husbands and 10 women with non-migrant husbands. The participants were selected purposively from a list of mothers with children 0–6 years of age. On the other hand, 35 IHPs were selected based on recommendations by the institution for which they worked and on the basis of demographic characteristics (age, sex and education), service delivery (type of practice and average monthly patients) and geographic location (deltaic and non-deltaic). During the selection of the stakeholders we aimed to achieve maximum variation.

The first step of the application of PSNA involved 2 months of general ethnographic observations, which allowed us to understand the social and physical context of the study area and its implications for the social networks of mothers as well as IHPs. This period was also helpful for rapport establishment with the villagers, local leaders, NGOs, community-based organisations and other important members of the village.

After briefing the mothers/IHPs about the objective and process of the study, a blank sheet of paper was given to each mother or IHP (Ego) to draw their personal network map. The procedure started with asking both the mothers and the IHPs to put themselves at the centre of the paper and then to identify all those individuals from whom they receive support (in relation to child care) in their personal and professional life. Personal life in this case captured the aspects of social, material and cognitive support, while the professional life captured economic, leveraging and skill-building support. The person’s name was then written on a piece of coloured paper; this was done by the researcher if the respondent was illiterate or preferred not to write. The respondent was then asked to affix the coloured paper to the large sheet. The distance between the two names provided a sense of physical or mental closeness between them and the support. Thereafter, the researchers asked the respondent to describe who this person was and the nature of help they provided. The researchers then asked them to name another person, whose name they wrote on another coloured piece of paper, and asked the respondent to affix it to the white sheet. They repeated the process until the respondent reported that there were no other people who provided them with help. Respondents were then given a pile of dried chickpeas and were asked to heap chickpeas on the name of each person who provided them support and who was now listed on the white sheet. The size of the heap matched the frequency of support and corresponded to a five-point scale (1, very frequent; 2, frequent; 3, sometimes; 4, rare; 5, never) on the basis of frequency of receiving support. Although this method did not allow precise measurement of the degree of support, it nevertheless allowed us to estimate the amount of support received from different stakeholders.

The final stage encompassed another set of in-depth interviews regarding the dynamics and pattern of the network, nature of ties and significance of each of the connections. They were asked to explain why the network was like that and how important the particular member is and why. If it was a very weak relationship, they were asked to explain why the relationship was weak. For example, the mothers said there was no connection with women self-help groups, which is a leveraging node that would be helpful to support them in accessing childcare resources; they said that being in a group was a man’s job. Absence of such leveraging ties may have an effect on the decision-making process of child care seeking and economic independence of the respondents as these kind of organisations are beneficial in terms of providing skills building and employment opportunities and developing self-esteem in the Indian context. In another example, on a positive note, the most important player in the mothers’ network was the IHPs, with whom every mother had strong ties. Respondents stated that, for minor to major children’s ailments, they unquestionably trust IHPs. When asked to explain this relationship, the mothers stated four main reasons, namely proximity, round-the-clock availability, treatment and medicines on credit, and belonging to the same community. They also stated that they preferred depending on the IHPs for treatment and referral, if necessary.

### Outcome of the engagement

#### Awareness

The aim of the engagement was to identify linkages between the demand and supply side stakeholders and to understand the pattern of relationships and gaps within the network. This was to facilitate the achievement of the project’s overarching goal of intervening knowledge into the system through different stakeholders. PSNA gave a significant understanding of how the system actors are connected with each other. The mothers are strongly connected with the IHPs and the IHPs were strongly connected with the private qualified providers, pharmaceutical companies and nursing homes, but weakly connected with the formal healthcare system and mainly with the frontline health workers. This information was presented through policy and research briefs, used to explain gaps within the network of the demand and supply side stakeholders of the Sundarbans’ health system, and to highlight areas where more collaboration was required.

Hence, the PSNA provided guidance on how to use the different actors to communicate specific types of information. This information was important for enhancing the existing connection between the stakeholders (both supportive and leveraging) so as to provide more effective service delivery to the regions where childcare is significantly affected by severe geo-climatic challenges.

### PIPA

#### Purpose of the engagement and why PIPA was used

We used this method to understand the key players involved in the Uganda FHS project (MANIFEST) as well as their importance and influence on maternal and newborn issues. Furthermore, we assessed the extent to which stakeholders felt the project had succeeded in addressing its objectives, in addition to identifying additional action that needed to be taken in order to achieve success. The latter included the identification of stakeholders that had not been involved and yet could be crucial in the implementation and sustainability of the project. Finally, the participatory nature of the project made PIPA a suitable tool for engaging stakeholders.

### Process of engagement

#### Participants

Two rounds of data collection were performed in each of the three districts. The first round was took place approximately 1.5 years after the MANIFEST project implementation had started and the second was held at the end of implementation. The implementation of PIPA was carried out during two different regularly scheduled stakeholder meetings for the project so as to reduce the costs of holding a meeting specifically for PIPA. The first round of data collection was performed over 10 dissemination meetings held in each of the three districts. Each dissemination meeting had approximately 60 participants, including sub-county chiefs, health workers, local political leaders, religious leaders, community development officers, VHT members, leaders of local NGOs and local community leaders. The second round of PIPA was conducted during sub-county review meetings (*n* = 10) with a smaller number of participants (*n* = 21) who met every quarter to review the progress of the project. They were all members of the MANIFEST sub-county implementation committee meeting. All those who attended this meeting had also been invited to the dissemination meeting where the first round of PIPA data collection was undertaken. Details of the number of PIPA group meetings held and the participants per group are provided in Table 1.

**Table 1:** Composition of PIPA group meetings

First Round of PIPA data collection			
	Kamuli	Pallisa	Kibuku
No of dissemination meetings	2	4	4
Average no of participants per dissemination meeting	60	60	60
No of PIPA group meetings	2	4	4
No of participants per PIPA group	8–10	8–10	8–10
Second round of PIPA data collection			
No of sub-county review meetings	2	4	4
Average no of participants per sub-county meeting	21	21	21
No of PIPA group meetings held	2	2	2
No of participants per group	5–7	5–7	5–7

#### Preparation and resources required

The resources required for the PIPA meeting included a venue that could allow free interaction, flip charts for recording responses, markers of different colours to allow differentiation of terms, and a team of facilitators and note-takers who understood commonly spoken languages. The sub-county provided the venue for the meetings. The district health team and the sub-county leadership mobilised participants for the meeting.

#### Training of facilitators

The Makerere university team and the district team conducted the facilitation of the PIPA sessions. The Makerere University team underwent four half-day trainings prior to the PIPA meeting. The first two trainings took place approximately 1 year before the PIPA data collection by external FHS partners based at Johns Hopkins University and the Institute of Development Studies. The initial training was mainly knowledge based and focused on providing the researchers with an understanding of what PIPA is and how it is applied. The second training focused mainly on the practical aspects of how to apply PIPA. During the third training, the Makerere University team discussed what PIPA was and how it is commonly used, questions that were to be answered using PIPA, and practical illustrations of how the network mapping was to be undertaken. The fourth round of training was performed after the first round of data collection. Hence, it provided an opportunity to recap how to apply PIPA and to reflect on challenges faced during the first data collection and possible solutions. One of the challenges noted was that different teams understood some concepts and instructions differently and therefore also implemented them differently. For example, some facilitators found it difficult to explain the difference between importance and influence of stakeholders, and therefore they only captured information on influence. Hence, for the second round, a more detailed guideline was developed for implementation of the data collection, outlining what was to be done and how. Secondly, definitions of key terms were agreed upon. The team also discussed how to improve time management because it was noted that this had been a problem for some of the teams during the first round of data collection. The team already had experience in facilitating group discussions; therefore, this was not included in the training package.

#### Application of the method

As mentioned earlier, PIPA often involves five distinct steps (construction of problem trees, visioning, developing network perspectives, defining an outcome logic model and an impact model). The decision to use PIPA was made midway through the project; by then, stage one of the PIPA process, which requires construction of problem trees, had been implemented (during the design phase of the project) using participatory techniques. Additionally, the use of an end-of-project survey to evaluate the project’s success had been planned and outcome and impact logic models had been developed. Consequently, the team modified their application of PIPA by doing a recap of stage one, then focusing mainly on stage two (visioning) and three (developing network models), and assessing outcomes and impacts from the perspectives of the stakeholders based on their own criteria.

The first round of PIPA started with a plenary session meeting during which a recap of the problems that led to the intervention were discussed. The group did not identify any additional problems. The MANIFEST project had identified these problems using participatory approaches during its design phase through a series of meetings that included workshops and focus group discussions with several stakeholders. Thereafter, the participants were divided into groups and only one of the groups went through the PIPA process (Table 1), while the other groups discussed other questions that were of interest to the project. The group that was involved in PIPA was guided through the questions and activities that were to be performed.

The second step of PIPA involved visioning; during this stage, the participants were asked to describe what successful implementation of the different aspects of the project would look like. Some of the responses from the group discussions are captured in Box 1.

Box 1 Perceptions about how success will look like

**Table:** 

• When we hear of no maternal and child deaths
• When traditional birth attendants close shop
• When we see an increase in fourth antenatal care attendance
• When all women are delivering at health facilities
• When mothers are well prepared for delivery
• When health workers have duty rosters
• When health workers are present on duty at any time
• When drugs and other supplies are available throughout the year
• When all health workers are accommodated at the health facilities

The third stage involved identification of the stakeholders and grouping them into similar groups. Thereafter, the participants discussed and ranked the stakeholders according to their level of involvement, importance and influence. The stakeholder network mapping was performed during the first PIPA meeting (before the Manifest programme), during the programme and at the end of the programme. To reflect the network map at the end of the programme, participants were asked to draw what they thought the network map would look like if they were successful. In the fourth and last stage, the participants were asked to describe how they would know that they have achieved success. This was intended to be a qualitative form of measurement of success rather than a logic outcome or impact model. This was based on the qualitative measures, which they had proposed during the first session of PIPA (Box 1).

**Fig. 2:**
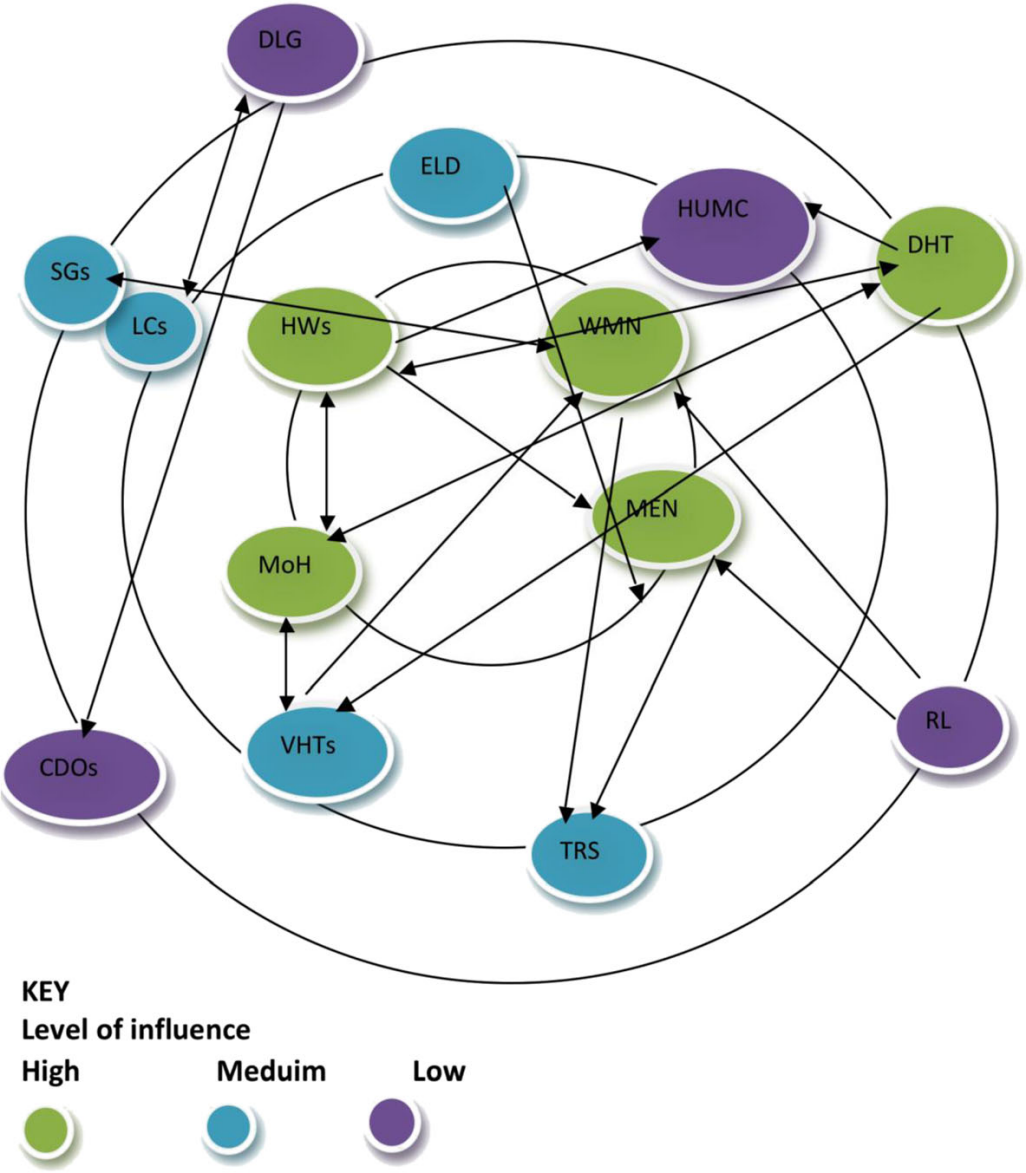
Network map before MANIFEST (Pallisa, Sept 2014). List of stakeholders: (1) men, (2) women (WMN), (3) transporters (TRS), (4) Saving Groups (SGs), (5) village health teams (VHTs), (6) local council leaders (LCs), (7) religious leaders (RL), (8) health unit management committee (HUMC), (9) chiefs (CHFs), (10) elders (ELD), (11) health workers (HWs), (12) community development officers (CDOs), (13) district local government (DLG), (14) district health team (DHT)

**Fig. 3:**
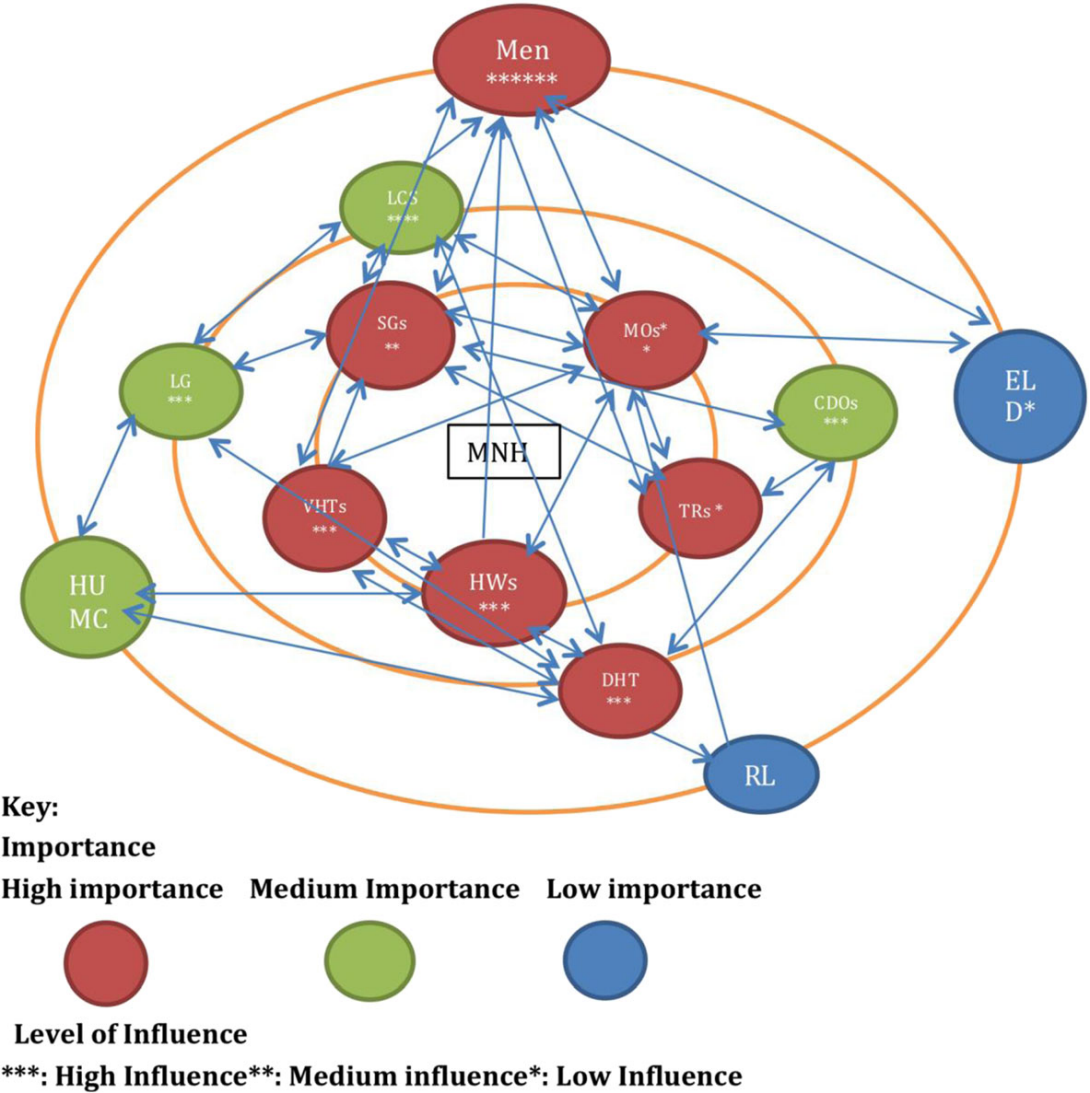
Network map at the end of MANIFEST (Pallisa, Dec 2015). List of stakeholders: (1) men, (2) women (WMN), (3) transporters (TRS), (4) saving groups (SGs), (5) village health teams (VHTs), (6) local council leaders (LCs), (7) religious leaders (RL), (8) health unit management committee (HUMC), (9) chiefs (CHFs), (10) elders (ELD), (11) health workers (HWs), (12) community development officers (CDOs), (13) district local government (DLG), (14) district health team (DHT)

The second round of PIPA was performed towards the end of the project and built onto that achieved during the first phase. The first stage of recapping was skipped since all the members in the sub-county team were aware of the initial problems that prompted the intervention. The second step of visioning was also skipped because it was not relevant at the end of the project. The third stage of network mapping was performed to reflect only the present situation rather than past, present and future. Two of the maps that were drawn at the first PIPA meeting, referring to the period before MANIFEST and at the end of the project, are displayed in Figs. 2 and 3.

During the second round, participants were asked to assess whether they had achieved success based on the indicators that they had identified earlier (Box 1). The quotes below represent some of the quotations that represented positive change and therefore success.

“*I went to get care at a health facility and a pregnant woman came for checkup. She was told that labour had started and therefore she had to be admitted. She immediately requested for a boda boda (motorcycle) to go home and pick a specific bag of her birth items, which she had prepared beforehand.*”

The improved birth preparedness depicted in the quotation was attributed to an improved culture of saving for MNH that was emphasised by MANIFEST through VHTs’ home visits and community dialogue meetings. In addition, health workers also conducted regular health education at facilities. It was also noted that maternal and newborn deaths had decreased, attributed to increased facility delivery as a result of increased awareness about the role of facility delivery in promoting the safety of newly delivered women and their newborns.

“*Deaths of mothers and their newborn babies are reported to be fewer these days as compared to prior to MANIFEST. In Bugulumbya two maternal deaths were reported this year.*”

However, it was noted that there were still areas that required further improvement. One of the areas mentioned was late arrival of health workers in some health facilities. Another area that was noted was male involvement. It was reported that some of the men give their wives money to prepare for birth but they do not attend the meetings held at the village level. This was captured in the quotations below.

“*Men do not escort their wives to the health facility during antenatal care and delivery, and do not attend meetings where MNH is discussed at the village level. Many men only buy materials for birth preparedness.*”

### Outcome of the engagement

#### Stakeholder involvement during PIPA

In terms of the composition of stakeholders, the first round had a more diverse group but the team was larger (8–10 people) and so it was more difficult to get consensus; the second round had a smaller team (5–7 people), but was less diverse.

During the reflection meeting, it was noted that the time that was required to conduct the PIPA meeting was rather long (5 hours) and so eventually some of the participants got tired. There was also a language barrier due to some participants not being able to understand English; therefore, there was a need to translate the questions into the local language.

#### Awareness

The participants were able to envision how they could expand their networks and resources by mentally thinking about the contributions that they could make to the project. After the first round of the PIPA meeting, the team reviewed the reports and identified actions and stakeholders that participants thought had been excluded or had not been utilised sufficiently.

#### Participation in project activities

During the research team meeting, suggestions were further considered and taken up if they were feasible and could fit within the existing resource envelope. It was also noted that some of the suggestions were already being implemented by the project, including suggestions such as use of the ‘super VHTs’ to support other VHTs and involvement of local political leaders in mobilisation for community dialogues.

The PIPA process and other project activities also provided an opportunity for the participants to discuss problems and to share how others had solved the problems. For example, late arrival to the facility was noted to be a problem; however, the involvement of sub-county leaders had helped improve this problem at another facility and therefore their involvement was recommended. The intervention of the sub-county chief in sorting out default from payment in saving groups was also noted to have reduced the problem of failure to pay debts.

The network maps drawn before the project and after the project also demonstrated that there was more participation by other stakeholders in issues related to maternal health. Before MANIFEST, groups such as saving groups and transporters were shown not to be closely involved to maternal health (in the outer circles); however, at the final meeting, they were all in the inner most circle (Figs. 2 and 3).

## Discussion

The discussion focuses on the appropriateness of using the tools, how the methods were adapted highlighting challenges and lessons learnt, and the outcomes of the engagement process.

### Comparison of the tools

Both PSNA and PIPA were found to have been appropriate tools to use for the purpose of the engagement. Application of PSNA with the combination of quantitative and qualitative tools allowed the team to understand not only the pattern of the network but also why and how this pattern emerged. Furthermore, it allowed the researchers to explore the structure of the networks from an etic angle, and the formative process of the networks from an emic angle. PSNA can play a key role in making visible the subjective relationships between actors and how these relationships can hinder or facilitate the flow of information and the effectiveness of an organisation or intervention [18, 21, 32]. Such information can be particularly useful for identifying appropriate knowledge brokers for different target audiences [21]. Similarly, PIPA also aided the identification of key stakeholders involved in maternal health. PIPA allowed more interaction between the stakeholders and thus promoted the sharing of positive practices and experiences and opened up opportunities for learning and further involvement in the project.

Further reflections about PIPA showed that the method is comprehensive in terms of its coverage and that it can cover several issues ranging from an understanding of key problems, identifying solutions to the problems, and choosing how to implement the solutions to evaluating progress with implementation and achievement of outcomes. However, this comprehensive coverage also means that implementing the entire method as often described [25] can be quite long (several days). Henceforth, in this particular participatory project, only aspects of the tool that would add value to the project were selected. This flexibility in the application of PIPA is therefore a strength. Implementation of the PIPA process at different time points is useful and allows the team to capture progress in implementation as seen from the perspective of its stakeholders over time. The first PIPA session in this case was implemented mid-way through the project and the second at the end. The project team noted that it would have been more beneficial to have implemented it at the beginning, middle and end of the project. Similarly, implementation of PSNA more than once would have allowed the team to analyse changes in the network structure after the implementation of the knowledge intervention.

The time required to conduct PSNA will vary with the objective of the study, funding resources and the study design; however, on the whole, the participatory nature requires more time to allow adequate interaction. Although it covers interrelationships at a specific time, because of the dynamic nature of social networks, there is constant change, which is also prone to external shocks like natural hazards, innovations or new interventions or socio-political movements. Consequently, the method has to be performed several times if one is interested in capturing such changes. Exploration of the social network of a household or community at a given point in time would therefore take a shorter time than exploration of changes in a network over time, which could include entry and exit of different actors within the system. Other challenges that have been identified in using SNA include difficulty in capturing the dynamics of the system and specifying boundaries.

### Lessons learned

Several lessons were learnt while implementing PIPA and PSNA. It was noted that using a language which the participants understand clearly was important for ensuring that participants understood the questions and to facilitate free discussion and participation. Secondly, we noted that participatory research projects allow close engagement with participants, with opportunities for feedback and reflection of the local problems and local solutions proposed; this is very similar to what is obtained through a PIPA method. This method can therefore be particularly suitable for projects that have limited engagement with their stakeholders because it would promote their level of engagement. Whereas the team that used PSNA piloted the tools, the team that used PIPA did not. The PIPA team noted that a longer period with piloting of the tools would have improved outcomes of the PIPA process. This therefore illustrates the importance of adequate preparation of teams that will engage in the use of new methods rather than the use of quick training approaches. Adequate preparation of the stakeholders for the engagement process is also important [4]. In the MANIFEST programme, it was assumed that all the stakeholders who were invited for the PIPA meeting were aware of the programme since it was being implemented in the district and many of them were active participants; therefore, no additional preparation took place. Similarly, in the Sundarbans, researchers were familiar with the settings and had good rapport with the stakeholders. Furthermore, the India team performed 2 months of ethnographic observations to build rapport with the local stakeholders and allow familiarisation with local contextual issues.

### Limitations

The methodological limitations of this paper include an inability to objectively measure the achievements of the outcomes identified in the conceptual framework and the use of the researchers implementing the project themselves as facilitators of the PIPA meetings, which may have led to some desirability bias where the respondents provide answers that they feel are expected by the facilitators. However, we believe that the long standing relationship between the researchers and the participants at the PIPA meetings may have led to the development of trust and therefore freedom of expression of both positive and negative opinions. Another limitation of our work was that stakeholders’ feedback from the PSNA was limited to a particular timeframe during the method implementation and not throughout the project intervention period. Consequently, some benefits of PSNA could have been missed. Similarly, the PIPA described in this paper did not concentrate on all the five major aspects of PIPA and so some lessons could have been missed. Furthermore, we were not able to assess the full impact of PSNA on the implementation of the knowledge intervention since the required data had not been collected. Finally, the outcomes of the engagement observed during the second PIPA meeting cannot be attributed only to PIPA because the stakeholders who participated where already involved in other participatory activities within the project.

## Conclusions

The use of PSNA and PIPA were appropriate for the purposes for which the projects wanted to engage with the stakeholders. While the India team was interested in identifying interrelationships between its demand and supply side stakeholders, the Uganda team was particularly interested in identifying the different roles played by its stakeholders and identifying mechanisms of collaborating more closely with them. Although in both cases the researchers felt that the outcomes of the stakeholder engagement were achieved, a more objective evaluation of the achievement of these outcomes is recommended in future research. The processes that were considered critical for successful application of the tools and achievement of outcomes included training of facilitators, language used during the facilitation, the number of times the tool is applied, length of the tools, pretesting the tools, and use of quantitative and qualitative methods. Finally, PIPA had a higher potential for promoting collaboration between the stakeholders, likely because it allowed more interaction between the stakeholders than the PSNA.

## Open peer review

Peer review reports for this article are available in Additional file 1.

## Additional file

Additional file 1: Open peer review reports. (DOCX 23 kb)

## Abbreviations

FHS: Future Health Systems
IHP: informal healthcare provider
MNH: maternal and neonatal healthcare
NGO: non-governmental organisation
PIPA: participatory impact pathway analysis
PSNA: participatory social network analysis
SNA: social network analysis
VHT: village health team
