# Supplementary material for: Engaging stakeholders: lessons from the use of participatory tools for improving maternal and child care health services
Source: Health Res Policy Syst. 2017 Dec 28;15(Suppl 2):106. doi: 10.1186/s12961-017-0271-z (PMC5751791; doi:10.1186/s12961-017-0271-z)
Supplement: Additional file 1: — Open peer review reports. (DOCX 23 kb) [file 12961_2017_271_MOESM1_ESM.docx]

**Title:** Engaging stakeholders: Lessons from the use of participatory tools for improving Maternal and Child Care Health Services

**Reviewer 1:** Susan B. Rifkin

**Reviewer’s report**

**Major compulsory revisions**

1. I think that the title should read: Engaging stakeholders: Lessons from the use of participatory tools for improving Maternal and Child Care Health Services. I think the existing title is too complicated and that also the clear link for MCH services should be put up front.
2. I think more needs to be said in the introduction about participatory approaches. A paragraph of why they are being promoted and the difference between their use as motivation for making “communities” do what professionals think best and empowerment where “communities” begin to take ownership of interventions needs to be identified and briefly discussed.
3. In the abstract I think it should be noted that in conclusion this research provides lessons for using these tools. This is a valuable part of the research and needs to be highlighted.

**Minor essential revisions**

1. In the section entitled ‘Overview of the tools and the context in which they were applied” it would be better to describe the tool first and then the context. It is easier for the reader to see the possible application of the tool if presented in this order as suggested in the title of this section.
2. In the “Discussion” I would change the order of presentation and give each section a subtitle: 1. Comparison of Tools 2. Limitations 3. Lessons Learned Under the last section it might be better to include the sentence in the Conclusion which states what did work

**Declaration of competing interests**

I have no conflict of interests.

**Reviewer 2:** Juliet Nabyonga Orem

**Reviewer’s report**

**General comments**

This is well written paper. It is good to see a good application of these participatory approaches in engaging beneficiaries in research, problem identification and forging solutions. The data are sound, discussion and conclusion well supported by data.

The writing is acceptable.

**Minor essential revisions**

Introduction:

1.	Please check again the details regarding Arnstein’s levels of stakeholder participation; you seem to have “second” twice.

2.	Page 6 – In reference to 4 – 6 stakeholders and relating this to the definition of stakeholders as these may be individuals, organisation, institutions. Please make it explicit that these were individuals within institutions or otherwise. If these are individuals within institutions/organisations, state how these were selected, was it based on the recommendation of the institutions/organisations? My imagination is that this was at two levels

1) you identified institutions/organisations at the beginning of the study and 2) within identified institutions/organisations an individual to participate in the research was selected and these are the ones participating in the SNA and PIPA discussions.

In case of local governments like the case of Uganda, it could be by designation

Make this explicit.

3.	Note that individuals may hold different views on specific issues other than the institutions/organisations they represent. I think this needs to be stated.

4.	You mention that PIPA was conducted alongside scheduled regular meeting implying that this process was additional to other processes (this is stated on pg 7). How did this compromise the quality of the engagements?

Methods:

5.	To a reader, the reflection meetings seem a bit confusing, how many were they? you state they were 3 in the first sentence, you also state that each country team held a reflection meeting (after reference 11), was this one of the 3 or it is additional making it 4? Is the reflection meeting after reference 11 only for researchers?You already have one for researches in the second sentence.

6.	I find the conceptual framework simplistic assuming a linear relations ship and not acknowledging the role of the broader context and external influence on these processes. The content presented on pg 20 para 2 can also be included here to strengthen the CF. Acknowledging external shocks, difficulty in capturing dynamics of both the systems and social stakeholder interactions and positions, boundaries. At least two sentences can be added to this effect.

Results:

7.	Page 10 – under the section on Application of the method.

You mention that 20 mothers were selected randomly. The last sentence on this para states that selection was aimed to get maximum variation. How can you ensure maximum variation in random selection? Why then did you not consider purposive selection?

8.	Page 11, it is OK for such a study as this to use this approach of looking a heaps of chickpeas and infer derive rankings but we also need to acknowledge that the boundaries of such derived rankings are grey because you cannot ascertain the sizes of heaps with certainty.

9.	Page 13, please say something regarding the nature of district facilitators, you mention that Makerere team was trained. Were the district facilitators social scientists, were they trained, were they part of the district leadership in which case they would in away influence responses? In Uganda, the political challenges impact the responses given, was this an issue especially when district teams are facilitators?

10.	Pages 13 – 14: you state that there was confusion between “importance” and “influence” in the first round of data collection implying that the first set of data could not be fully comparable to the second set of data. How was this reconciled?

11.	Page 14 – application of the method. Authors state that the construction of the problem trees was undertaken during the design phase of the project and this was before the research team was trained in PIPA. There was a recap in the first phase of data collections, were there differences in problems identified requiring modifications in the implementation of the project? This needs to be stated.

12.	Last para pg 14: there is a statement that “while the other groups went through other activities” were these PIPA activities or other project issues?

13.	Page 15 “The third stage of network mapping was done to reflect only the current situation rather than past, present and future” Is there a difference between “current” and “present”?

14.	Box 4: Men do not escort “their”

**Declaration of competing interests**

I declare no competing interests

**Author’s response to reviews**

**Reviewer 1:** Susan B. Rifkin

**Major compulsory revisions**

1. I think that the title should read: Engaging stakeholders: Lessons from the use of participatory tools for improving Maternal and Child Care Health Services. I think the existing title is too complicated and that also the clear link for MCH services should be put up front.

**The title has been changed as advised**

2. I think more needs to be said in the introduction about participatory approaches. A paragraph of why they are being promoted and the difference between their use as motivation for making “communities” do what professionals think best and empowerment where “communities” begin to take ownership of interventions needs to be identified and briefly discussed.

**Thank you for this advise this has been done. Pg. 4 - See paragraph four in the introduction.**

3. In the abstract I think it should be noted that in conclusion this research provides lessons for using these tools. This is a valuable part of the research and needs to be highlighted.

**This has been done.**

**Minor essential revisions**

4. In the section entitled ‘Overview of the tools and the context in which they were applied” it would be better to describe the tool first and then the context. It is easier for the reader to see the possible application of the tool if presented in this order as suggested in the title of this section.

**Thank you for this suggestion. This has been done see page 5 and 6**

5. In the “Discussion” I would change the order of presentation and give each section a subtitle: 1. Comparison of Tools 2. Limitations 3. Lessons Learned Under the last section it might be better to include the sentence in the Conclusion, which states what did work.

**We have added the subtitles and changed the order of some aspects of the discussion, however we have put the limitations of the paper at the end of the discussion. Pg. 20**

**The last statement had been included earlier in the discussion see paragraph one in the discussion last sentence. Pg. 19**

**Reviewer 2:** Juliet Nabyonga Orem

**Minor essential revisions**

Introduction:

6. Please check again the details regarding Arnstein’s levels of stakeholder participation; you seem to have “second” twice.

**This has been adjusted.**

7. Page 6 – In reference to 4 – 6 stakeholders and relating this to the definition of stakeholders, as these may be individuals, organisation, and institutions. Please make it explicit that these were individuals within institutions or otherwise. If these are individuals within institutions/organisations, state how these were selected, was it based on the recommendation of the institutions/organisations? My imagination is that this was at two levels 1) you identified institutions/organisations at the beginning of the study and 2) within identified institutions/organisations an individual to participate in the research was selected and these are the ones participating in the SNA and PIPA discussions.  In case of local governments like the case of Uganda, it could be by designation  Make this explicit.

**Thank you for pointing this out we have made this explicit. See page 10.**

8. Note that individuals may hold different views on specific issues other than the institutions/organisations they  represent. I think this needs to be stated.

**We do agree that individuals may hold different views on specific issues other than the institutions/organisations they  represent. However we have not stated this because we feel that it was not a major issue in this paper. During analysis we did not encounter data that reflected issues that were highly controversial.**

9. You mention that PIPA was conducted alongside scheduled regular meeting implying that this process was  additional to other processes (this is stated on pg. 7). How did this compromise the quality of the **engagements?**

**We did this to reduce on the expenditure costs of conducting PIPA. However we tried to ensure that we covered everything that was necessary, so we do not think that it affected the quality of the engagements. Since we had several participants, we were able to divide them so that some of them participated in the PIPA meetings while others participated in the other project related discussions.**

Methods:

10. To a reader, the reflection meetings seem a bit confusing, how many were they? You state they were 3 in the first sentence, you also state that each country team held a reflection meeting (after reference 11), was this one of the 3 or it is additional making it 4? Is the reflection meeting after reference 11 only for researchers? You already have one for researches in the second sentence.

**We have revised this to make it more clear. Page 7 first paragraph in methods section.**

11. I find the conceptual framework simplistic assuming a linear relations ship and not acknowledging the role of the broader context and external influence on these processes. The content presented on pg. 20 para 2 can also be included here to strengthen the CF. Acknowledging external shocks, difficulty in capturing dynamics of the systems and social stakeholder interactions and positions, boundaries. At least two sentences can be added to this effect.

**We have acknowledged the above observation about the framework.**

Results

12. Page 10 – under the section on Application of the method.  You mention that 20 mothers were selected randomly. The last sentence on this para states that selection was aimed to get maximum variation. How can you ensure maximum variation in random selection? Why then did you not consider purposive selection?

**Thank you for this observation, we agree that this is more of purposive sampling than random sampling so we have changed this statement**

13. Page 11, it is OK for such a study as this to use this approach of looking at heaps of chickpeas and infer derive rankings but we also need to acknowledge that the boundaries of such derived rankings are grey because you cannot ascertain the sizes of heaps with certainty.

**This has been acknowledged. See page 11.**

14. Page 13, please say something regarding the nature of district facilitators, you mention that Makerere team was trained. Were the district facilitator’s social scientists, were they trained, were they part of the district leadership in which case they would in away influence responses? In Uganda, the political challenges impact the responses given, was this issue especially when district teams are facilitators?

**The district facilitators comprised of health workers and social scientists. We do not believe that they influenced the responses significantly because many of the issues discussed were not highly political, and when they related to the health sector it was often about local health facilities.**

15. Pages 13 – 14: you state that there was confusion between “importance” and “influence” in the first round of data collection implying that the first set of data could not be fully comparable to the second set of data. How was this reconciled?

**We were not able to reconcile this. However since the main purpose of this paper was not to measure the influence that stakeholders had we feel that this does not introduce any major methodological issues in this paper**

16. Page 14 – application of the method. Authors state that the construction of the problem trees was undertaken during the design phase of the project and this was before the research team was trained in PIPA. There was a recap in the first phase of data collections, were there differences in problems identified requiring modifications in the implementation of the project? This needs to be stated.

**No there were no differences. This has been stated. Page 15 paragraph one.**

17. Last para pg. 14: there is a statement that “while the other groups went through other activities” were these PIPA activities or other project issues?

**They discussed other questions. We have rephrased this sentence to indicate this. Page 15 paragraph one.**

18. Page 15 “The third stage of network mapping was done to reflect only the current situation rather than past, present and future” Is there a difference between “current” and “present”?

**We have changed the word current to present, since the meaning was the same.**

19. Box 4: Men do not escort “their”

**This has been corrected**
